# The Use of an Organo-Selenium Peptide to Develop New Antimicrobials That Target a Specific Bacteria

**DOI:** 10.3390/antibiotics10060611

**Published:** 2021-05-21

**Authors:** Phat Tran, Jonathan Kopel, Joe A. Fralick, Ted W. Reid

**Affiliations:** 1 Department of Ophthalmology and Visual Sciences, Texas Tech University Health Sciences Center, Lubbock, TX 79430, USA; phat.tran@ttuhsc.edu; 2 Cell Biology and Biochemistry, Texas Tech University Health Sciences Center, Lubbock, TX 79430, USA; Jonathan.kopel@ttuhsc.edu; 3 Department of Immunology and Molecular Microbiology, Texas Tech University Health Sciences Center, Lubbock, TX 79430, USA; Joe.fralick@ttuhsc.edu

**Keywords:** selenium, phage, phage display technology, phage library, glutathione, free radicals, superoxide, *Yersinia pestis*, *Escherichia coli*

## Abstract

This study examines the use of a covalently selenium-bonded peptide and phage that binds to the *Yersinia pestis* F1 antigen for the targeting and killing of *E. coli* expressing this surface antigen. Using a Ph.D.-12 phage-display library for affinity selection of the phage which would bind the F1 antigen of *Y. pestis*, a phage displaying a peptide that binds the F1 antigen with high affinity and specificity was identified. Selenium was then covalently attached to the display phage and the corresponding F1-antigen-binding peptide. Both the phage and peptides with selenium covalently attached retained their binding specificity for the *Y. pestis* F1 antigen. The phage or peptide not labeled with selenium did not kill the targeted bacteria, while the phage or peptide labeled with selenium did. In addition, the seleno-peptide, expressing the F1 targeting sequence only, killed cells expressing the F1 antigen but not the parent strain that did not express the F1 antigen. Specifically, the seleno-peptide could kill eight logs of bacteria in less than two hours at a 10-µM concentration. These results demonstrate a novel approach for the development of an antibacterial agent that can target a specific bacterial pathogen for destruction through the use of covalently attached selenium and will not affect other bacteria.

## 1. Introduction

Antibiotic resistance is rising to dangerously high levels in all parts of the world. New resistance mechanisms are emerging and spreading globally, threatening our ability to treat common infectious disease. According to the Centers for Disease Control and Prevention (CDC), each year in the US, over 2.8 million people are stricken with antibiotic-resistant infections, of which more than 35,000 die (https://www.cdc.gov/drugresistance/index.html, accessed on 20 May 2021). The plague is a zoonotic infection caused by *Yersinia pestis*, a Gram-negative bacillus. This bacterium has been the cause of three great pandemics of human disease in the 6th, 14th, and 20th centuries [1,2]. The plague is transmitted from rodents by fleas, which cause an abrupt onset of high fever, painful local lymphadenopathy, and bacteremia in human subjects. Septicemic plague can sometimes ensue from untreated bubonic plague or, de novo, after a flea bite [2]. Patients with the bubonic form of the disease may develop secondary pneumonic plague (also called plague pneumonia); this complication can lead to human-to-human spread by the respiratory route and cause primary pneumonic plague, the most severe and frequently fatal form of the disease [2,3]. Plague has been considered the second most dangerous bacteria after *Bacillus antharacis,* and it can potentially be used in biological warfare [2,4,5]. The World Health Organization reports 1000 to 3000 cases of plague every year, and the mortality rate is between 5% and 12%. In the US, an average of 10 to 20 cases of plague occurs each year, and the mortality rate is 14% (1 in 7). Therefore, there is a need for the discovery and development of new antibacterial compounds that would circumvent bacterial resistance mechanisms. 

In an attempt to design a new class of antibiotics that would not exhibit drug resistance, we utilized the element selenium. Selenium has been shown to function as a catalytic generator of superoxide radicals (O_2_^−^) from the oxidation of thiols. The catalytic attribute of selenium has been known for nearly five decades, but the pro-oxidative characteristics of selenide compounds were not elucidated until the 1990s [6]. Seleno-compounds are reduced by thiols, forming the selenide anion RSe^¯^. RSe^¯^ is the catalytic species that oxidizes thiols (glutathione in particular) to produce superoxide radicals, hydrogen peroxide (H_2_O_2_), and a putative thiyl radical [7]. With the elucidation of the human genome and the known UGA codon for selenocysteine, 25 human seleno-containing structural proteins and enzymes are believed to exist. Hence, selenium is nutritionally essential for humans, and seleno-proteins play critical roles in reproduction, thyroid hormone metabolism, DNA synthesis, and protection from oxidative damage and infection [8]. 

Several studies have shown that selenium compounds such as thiaselenazoles, dithiazoles, and selenium–platinum complexes are effective antimicrobial and antiviral agents [9,10,11,12,13]. These agents allow for both narrow- and broad-spectrum antimicrobial and viral activity at micromolar concentrations with limited toxicity [9,10,11,12,13]. Furthermore, these agents are effective against multidrug-resistant bacteria and the formation of bacterial biofilms [9,10,11,12,13]. An ideal antibacterial drug would target the virulence mechanisms of bacterial pathogens and not be affected by existing resistance mechanisms in these bacteria [14]. One method for developing targeted antibacterial therapies is through the use of phage display technology. Using phage display technology, organo-seleniated peptides can be developed to target a specific receptor on bacteria to deliver the selenium to bacteria without damaging healthy cells. As a test case, in this study, we employed phage display technology to obtain peptide sequences that have a high affinity/specificity for the F1 antigen of *Yersinia pestis* for the development of seleno-peptide antimicrobials that target a specific bacteria. The killing mechanism of selenium is due to its ability to catalytically generate superoxide radicals, which can be seen in the diagram below.


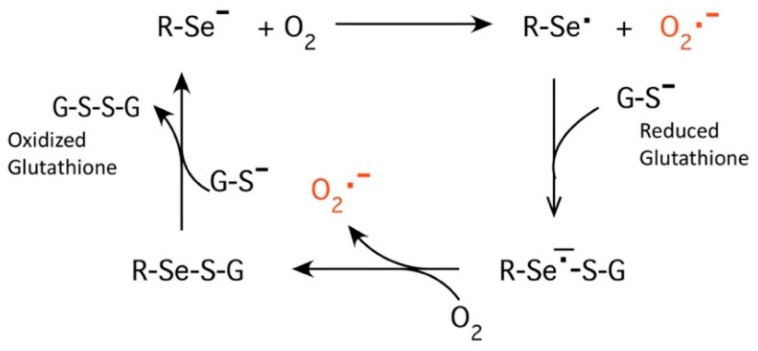


As seen in the diagram, ionized selenium is attached by a covalent bond to an organic compound (R-Se^-^). This ionized form donates an electron to oxygen, resulting in a selenium radical, R-Se *, and superoxide, in red. The selenium radical then reacts with reduced glutathione, G-S^−^. The resulting seleno-sulfide radical then reacts with a second oxygen molecule to form a second superoxide. An additional reduced glutathione then donates an electron to reform the original ionized organo-selenium and produce oxidized glutathione, G-S-S-G. This shows the ability of selenium to reduce oxygen to form superoxides while oxidizing glutathione in a catalytic mechanism. Glutathione is present in all body fluids. 

## 2. Results

### 2.1. Biopanning against the Y. pestis F1 Antigen

In order to select phage-display peptides against the *Y. pestis* F1 antigen, we employed the Ph.D. 12 phage display library, which has a diversity of 2 × 10^9^. We isolated 15 phages that could bind the purified F1 antigen with high affinity.

### 2.2. Characterization of Y. pestis F1 Antigen-Specific Display Phage

**Figure 1:**
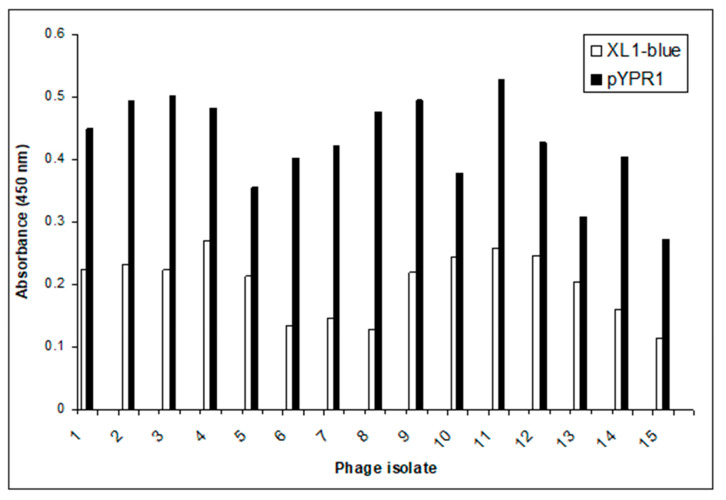
*Y. pestis* F1 antigen phage Spun-Cell ELISA. The binding ability of each phage isolated (*x*-axis) after the 3rd round of biopanning against the *Y. pestis* F1 antigen was tested through a Spun-Cell ELISA, comparing A450 nm values (*y*-axis) of the *E. coli* XL1-blue/pYPR1 strain and the parent *E. coli* XL1-blue strain. As seen, each phage isolate tested bound to the pYPR1 cells at a higher level than the XL1-blue cells. Phage isolate #8 showed the greatest difference between specific binding (i.e., binding to *E. coli* expressing F1 antigen) and nonspecific binding (binding to *E. coli* not expressing F1 antigen) at A450 nm, and it was selected for further characterization.

After the completion of the biopanning against the purified *Y. pestis* F1 antigen, 15 phage clones were randomly selected and amplified according to their specificity/affinity. This was analyzed by their ability to bind to the recombinant F1 antigen expressed on the surface of *E. coli* (XL1-blue/pYPR1) using a screening ELISA assay. The results can be seen in Figure 1. An initial Spun-Cell ELISA revealed that all 15 of the selected display phage clones showed preferential binding to the *E. coli* strain expressing the F1 antigen over the parent strain that does not express the F1 antigen.

The DNA from each of the selected phages was purified and sequenced by the Center for Biotechnology and Genomics Core Facility, Texas Tech University, Lubbock, TX. The deduced amino acid sequences of the peptide inserts are given in Table 1. From the DNA sequences, it can be seen that there appear to be some potential consensus sequences (i.e., putative binding motifs). Of particular interest is the 13 sequences containing a proline, where nine of these sequences had the proline located in the middle of the peptide. This appears unusual, as proline does not often occur in sequences since it is thought to cause an unconformity in the conformation of alpha-helical peptides. In most circumstances, the prolines would produce a confirmational change that would prevent a peptide from binding to its target. The presence of the prolines in the middle of the peptide may prevent significant conformational changes to the peptide’s ability to bind to its specific receptor or target. There were also a large number of Phe-Ser or Phe-Thr sequences (11/15) and Phe-Ser (Thr)-Leu (Ile) (7/15) in the peptides. In 3 peptides, the Phe-Ser (Thr)-Leu is followed by a basic amino acid. This analysis was confirmed using the Multiple Em for Motif Elicitation (MEME) tool (https://memesuite.org/meme/tools/meme, accessed on 20 May 2021) for peptide sequence comparison (Supplementary Figures S1–S4). Thus, these 15 clones showed a considerable amount of consensus despite being derived from a display phage library with a diversity of a minimum of 2 × 10^9^ different 12 amino acid sequences.

### 2.3. Antibacterial Activity of Seleno-Phage #8, Selected for Binding against E. Coli XL1-Blue/pYPR1

We hypothesized that the selenium-labeled phage could selectively bind to outer membrane proteins and destroy a specific bacterium through the generation of reactive oxygen species (ROS) on their surface. We investigated our hypothesis by testing the ability of the seleno-phage to selectively kill *E. coli* expressing the *Y. pestis* F1 antigen XL1-blue/pYPR1). Based on our results from the Spun-Cell ELISA assay (Figure 1), we selected phage #8 as our seleno-phage.

**Figure 2:**
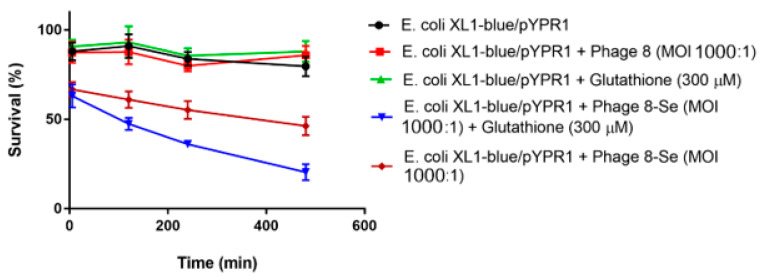
The effect of selenium-labeled phage #8 on the viability of *E. coli* XL1-blue/pYPR1. Survival of the *Y. pestis* F1-antigen-expressing *E. coli* XL1-blue/pYPR1 strain in the presence of selenium-labeled phage #8 at an MOI (multiplicity of infection) of 1000:1, with and without glutathione, at 150 or 300 µM concentration. The XL1-blue/pYPR1 strain was incubated with selenium-attached phage #8, with and without reduced glutathione (150 or 300 µM) in PBS. The control only had *E.* *coli* XL1-blue/pYPR1 in PBS. Other controls included XL1-blue/pYPR1 with either reduced glutathione or phage #8 in PBS. The experiments were carried out at room temperature for 8.5 h. After several time points, the aliquots of solutions were serially diluted and plated on LB agar supplemented with 100 µg/mL carbenicillin, and c.f.u. was determined after overnight incubation. Values represent the results from two independent experiments. Multiplicity of infection (MOI) is the ratio of phage to bacteria.

To perform the killing assay, *E. coli* XL1-blue and XL1-blue/pYPR1 cells were incubated with seleno-phage #8 in PBS at a multiplicity of infection (MOI) of 1000:1 at room temperature. Reduced glutathione was also added to determine if it had any effect on bacterial survival. Specifically, reduced glutathione at 150 and 300 µM was incubated with XL1-blue/pYPR1 cells without the phage to eliminate the possibility that reduced glutathione is toxic to cells. As shown in Figure 2, the *Y. pestis* F1-antigen-specific seleno-phage #8 killed *E. coli* expressing antigen F1 (XL1-blue/pYPR1), and the killing was increased in the presence of reduced glutathione. Phage #8 without selenium had no effect on either the *E. coli* strain expressing antigen F1 (XL1-blue/pYRP1) (Figure 2) or its parent (XL1-blue) (data not shown).

### 2.4. Antibacterial Activity of Seleno-Phage #8 on E. Coli (XL1-Blue/pYPR1) under Added Oxygen Condition

**Figure 3:**
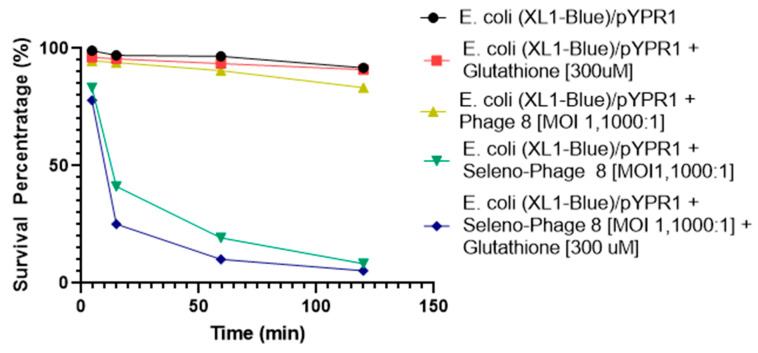
The effect of selenium-attached phage #8 on the viability of *E. coli* XL1-blue/pYPR1 under added oxygen. Survival of the *Y. pestis* F1-antigen-expressing *E. coli* XL1-blue/pYPR1 strain in the presence of selenium-labeled phage #8, an MOI (multiplicity of infection) of 1000:1, and 21% oxygen, with and without reduced glutathione (300 µM) in PBS. The control contained bacteria pYPR1 in PBS. Other controls included pYPR1 with either reduced glutathione or phage #8 in PBS. The experiments were carried out at room temperature for two hours. After several time points, aliquots of the solutions were serially diluted and plated on LB agar supplemented with 100 µg/mL carbenicillin and c.f.u. was counted after overnight incubation. Multiplicity of infection (MOI) is the ratio of phage to bacteria.

Given that 5 min elapsed before the number of bacteria at the first time point was determined, it was speculated that low oxygen tension in the media might contribute to a less robust generation of superoxide by the selenium catalysis in the later stages of these experiments. This is due to superoxide radical generation from the selenide anion, RSe^¯^, requiring the presence of oxygen [15,16,17,18,19,20]. We, therefore, tested the hypothesis by adding oxygen to the media to observe whether additional oxygen would enhance the antibacterial activity of the selenium-labeled phage. The experiments were performed with only one concentration of reduced glutathione (300 µM). The *E. coli* XL1-blue/pYPR1 cells were incubated with selenium seleno-phage #8 and exposed to oxygenation (21%). As shown in Figure 2, it took approximately 15 h to kill half of the bacteria without added oxygen; with additional oxygen, it only required approximately 3 h (Figure 3).

### 2.5. Antibacterial Activity of Seleno-Peptide #8 on E. Coli XL1-Blue/pYPR1 under Added Oxygen Condition

**Figure 4:**
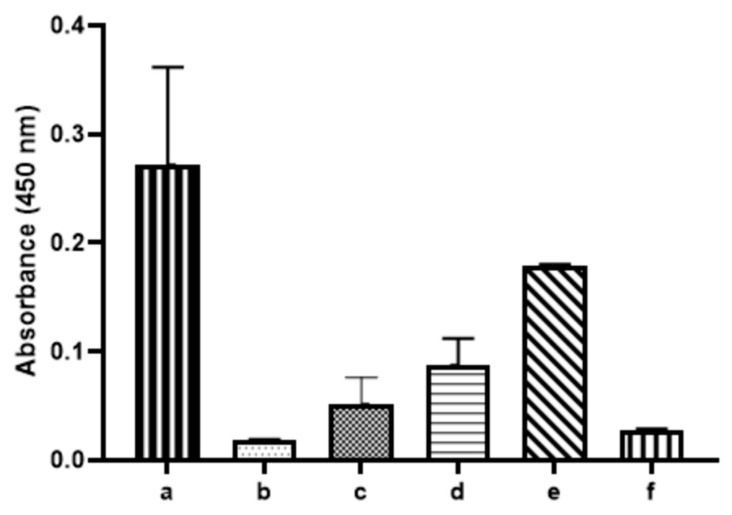
Competition of peptide #8 (1 μM) and F1 antibody (0.25 μg/mL) for the F1 antigen on *E. coli* XL1-blue/pYPR1 after incubation for 1 h at 4 °C; a. *E. coli* XL1-blue/pYPR1 cells incubated with F1 antibody; b. *E. coli* XL1-blue/pYPR1 alone; c. parent cells *E. coli* XL1-blue plus F1 antibody; d. *E. coli* XL1-blue/pYPR1 incubated with peptide #8 for one hour, and then the F1 antibody was added; e. *E. coli* XL1-blue/pYPR1 cells incubated with F1 antibody for one hour and then peptide #8 was added; f. *E. coli* XL1-blue cells incubated with peptide #8 and the F1 antibody. Error bars represent the mean ± SD of results from two independent experiments.

To examine if the free peptide could bind to antigen F1, we ran a competitive spun-cell ELISA against an anti-F1 antibody. As shown in Figure 4, peptide #8 was shown to compete with the F1 antibody for F1 antigen on *E. coli*/pYPR1 cells. We examined the ability of seleno-peptide #8 to kill *E. coli* expressing the F1 antigen. 

**Figure 5:**
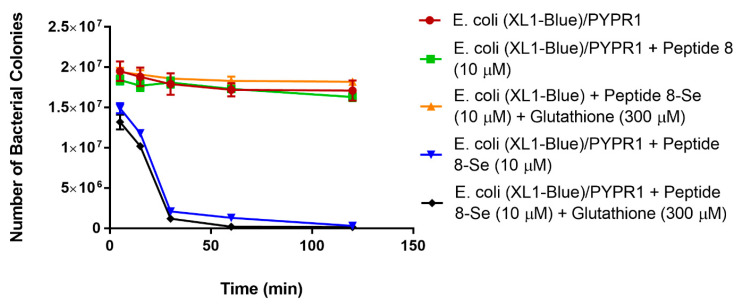
The effect of seleno-peptide #8 (10 µM) on the *E. coli* XL1-blue/pYPR1 strain, with or without glutathione, under added oxygen. One control contained only bacteria XL1-blue/pYPR1. Other controls included XL1-blue/pYPR1 with either reduced glutathione (300 µM) or peptide #8 (10 µM) without selenium. Values represent the mean from three independent experiments. *E. coli* (XL1-blue) is the parent strain without the plasmid for the F1 antigen.

**Figure 6:**
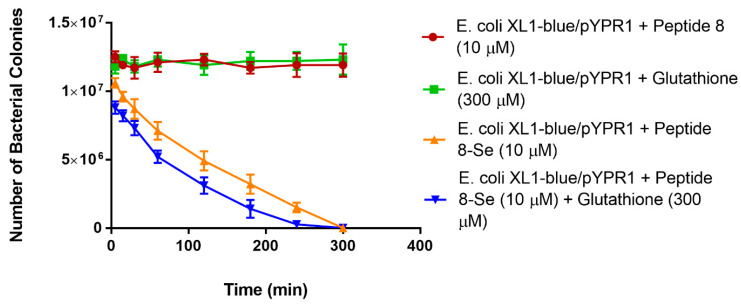
The effect of seleno-peptide #8 (10 µM) on the *E. coli* XL1-blue/pYPR1 strain, with or without glutathione, under added oxygen at 4 °C. Values represent the mean from three independent experiments.

As seen in Figure 5, the presence of added oxygen with seleno-peptide #8 killed over 90% of the bacteria expressing the *Y. pestis* F1 antigen in 1 h and most bacteria in 2 h at a 10 µM concentration. This concentration (10 µM) of peptide would have no toxic effects on humans. However, it is toxic to the targeted bacteria due to their limited antioxidant defense mechanisms to deal with locally concentrated superoxide radicals. Thus, a seleno-peptide has the potential to target specific bacteria and kill them without affecting other bacteria or cells in the host. It should be noted that this seleno-peptide does not kill the parent bacteria that do not express the F1 antigen on their surface. We also carried out the killing assay under nongrowing conditions at 4 °C (Figure 6). The cells were treated with seleno-peptide #8 (10 µM), either with or without glutathione. What was observed was that the peptide with selenium could kill the bacteria at 4 °C. It was slower than at 37 °C, but these were nongrowing bacteria. This is a temperature where normal antibiotics do not work due to the inability to enter cells. Overall, this experiment also demonstrates that the seleno-peptide works on the outside of cells.

It is important to note that the *E. coli* (XL1-blue) parent strain, without the plasmid for the F1 antigen, shows no killing by peptide-8 with selenium. This indicates that the selenium peptide is only able to kill bacteria that express the F1 antigen on their surface.

## 3. Discussion

The known virulence factors of *Y. pestis* are encoded on its chromosome and three plasmids [2,21,22,23,24,25]. The major virulence factors of *Y. pestis* are located in the outer membrane and enable the bacteria to directly translocate into host cells through a type 3 secretion system (T3SS) [2]. One of these virulence factors is the major capsular antigen fraction 1 (F1), which is essential for protecting the organism against phagocytosis [3,26]. This unique F1 antigen, which is coded by a 100-kb plasmid of *Y. pestis*, is antiphagocytic and essential for full virulence in some animal species [25,27,28]. In addition, *Y. pestis* exhibits diminished capsular expression at normal room temperature but switches to full expression when grown at 37 °C [3,28]. The F1 protein is antigenic. Specifically, sera from convalescent plague patients and experimentally infected mice showed a majority of antibodies against the *Y. pestis* F1 antigen [29,30]. *Y. pestis* can interfere with the immune system in humans by injecting the F1 protein into macrophages [25]. However, F1 can also function as a protective immunogen [31,32]. As little as 1 µg of recombinant F1, cut from an acrylamide gel, protected mice against 10^5^ parenterally administered virulent organisms [17]. Furthermore, mice immunized with live recombinant *Salmonella* strains expressing F1 were protected against a parenteral plague challenge. The fact that F1 antibodies can protect against *Y. pestis* indicates that the F1 antigen must be expressed in vivo and would make a good drug target [33,34,35,36].

Drug-resistant strains of *Yersinia pestis* have been reported [14]. Two plasmid-mediated multidrug-resistant strains of *Yersinia pestis* were isolated in Madagascar [37]. A recent study found two strains that were highly resistant to ampicillin, chloramphenicol, kanamycin, streptomycin, sulfonamides, tetracycline, and minocycline [38]. Specifically, Wong et al. studied the susceptibility of 92 strains of *Y. pestis* to various antimicrobial agents and found that only cefixime, ceftriaxone, TMP/SMO, and trovafloxacin were effective against the *Y. pestis* strains [39]. This occurrence of microbial resistance has made many excellent, potent, and inexpensive antibiotics almost obsolete [40,41,42].

Overall, antibiotic resistance is rising to dangerously high levels in all parts of the world. New resistance mechanisms are emerging and spreading globally, threatening our ability to treat common infectious disease. It is our premise that small Se-tagged peptides (STPs) that target a specific pathogen will be effective in treating antibiotic-resistant infections without affecting the normal flora of the patient, thus diminishing selection for resistance. In this study, we have shown that a selenium-conjugated display phage and a Se-conjugated peptide that binds the F1 antigen of *Y. pestis* can kill *E. coli* expressing this antigen while sparing those that do not. It has also been shown that a Se-conjugated anti-erythrocyte antibody can lyse erythrocytes but not if the erythrocytes had been previously treated with the same antibody missing the Se tag (i.e., blocking the receptor) [43]. These results show that the selenium-conjugated macromolecule must bind to the targeted cell to kill it and supports our hypothesis that selenium-conjugated peptides can specifically kill targeted bacterial pathogens without affecting other normal bacterial flora.

The fact that the selenium-labeled phage and the Se-peptide at 4 °C could kill the targeted bacteria would indicate that the killing is from outside the bacteria. This would show that the selenium does not have to enter the bacteria and, thus, would not be susceptible to efflux pumping mechanisms by the bacteria. In addition, since the seleno-peptide could only kill bacteria expressing the targeted protein, it shows the selectivity of this type of antimicrobial.

## 4. Materials and Methods

### 4.1. Bacterial Strains, Media, and Reagents

*Escherichia coli* (*E. coli*) XL1-blue/pYPR1 expressing *Yersinia pestis* (*Y. pestis*) F1 from the cloned *caf* operon and purified *Y. pestis* F1 protein were described earlier [18]. The cloned plasmid was obtained from T. Schwan, Rocky Mountain Laboratories, Hamilton, Montana. The *E. coli* ER2738 strain and PhD—12 phage-display kit were purchased from New England Biolabs, Inc. (www.neb.com, accessed on 20 May 2021). The *Escherichia coli* XL1-blue parent strain was obtained from Stratagene (Stratagene, La Jolia, CA, USA). The *E. coli* were maintained at 4 °C in Luria Broth (LB) medium containing 30% (*v*/*v*) glycerol. Antibiotics were used at the following concentrations in the LB medium: 20 µg/mL tetracycline for *E. coli* ER2738 and 100 µg/mL carbenicillin for the expression of the *Yersinia pestis* F1 antigen in *E. coli* XL1-blue/pYPR1. In addition, the *E. coli* ER2738 strain was grown with shaking at 37 °C in LB medium or on LB agar supplemented with 20 µg/mL tetracycline. The *E. coli* XL1-blue parent strain was grown with shaking at 37 °C in LB medium or on LB agar. In contrast, the *E. coli* XL1-blue/pYPR1 strain was routinely grown with shaking at 37 °C in LB medium or on LB agar supplemented with 100 µg/mL carbenicillin to maintain the pYPR1 plasmid. *E.* *coli* ER2738 was used to grow the phage.

The LB medium contained 10 g/L Bacto-Tryptone (#211705, BD BioSciences, San Jose, CA), 5 g/L yeast extract (#212750, BD BioSciences, San Jose, CA, USA), and 5 g/L NaCl (#S271-3, Fisher Sci., Hampton, NH, USA). In contrast, LB/IPTG/X Gal Plates contained the basal LB medium with 15 g/L agar (#214010, BD BioSciences, San Jose, CA, SUA), 50 µg/L IPTG/XGal (#15529-019, Invitrogen, Carlsbad, CA, USA), and 40 µg/L XGal (#15520-018, Invitrogen). The LB agar was composed of the LB basal medium and 15 g/L agar. The blocking buffer contained 0.1 M NaHCO3 (pH 8.6), 5 mg/mL BSA, and 0.02% NaN3, which was filter-sterilized and stored at 4 °C. The PEG/NaCl solution contained 20% (*w*/*v*) polyethylene glycol–8000 and 2.5 M NaCl, which was autoclaved and stored at room temperature. The iodide buffer [10 mM Tris-HCl (pH 8.0), 1 mM EDTA, and 4 M NaI. The solution was stored at room temperature in the dark], TBS solution, [50 mM Tris-HCl (pH 7.5) and 150 mM NaCl. Autoclave, store at room temperature], and Agarose Top [per liter: 10 g Bacto-Tryptone, 5 g yeast extract, 5 g NaCl, 1 g MgCl_2_·6H_2_O, and 7 g agarose. The solution was autoclaved and dispensed into 50 mL aliquots, stored solid at room temperature, and melted in a microwave as needed] were used in the experiments.

### 4.2. Preparation of Cells for Infection

A single colony of *E. coli* ER2738 from a stock plate was grown in twenty milliliters of LB medium at 37 °C with shaking at 225 rpm overnight. Aliquots of these cells were used immediately for transfection after each round of biopanning.

### 4.3. Biopanning and Screening of Phage-Displayed Peptides against Purified Y. pestis F1 Antigen

To select specific peptide sequences with high *Y. pestis* specificity and affinity, we screened a random peptide filamentous (M13) phage display library (PhD-12) for their ability to bind to the pure F1 antigen. Biopanning was performed following the method described in the Instruction Manual (pH D.-12 phage display, New England Biolabs Inc.) using TBS [50 mM Tris-HCl (pH 7.5) and 150 mM NaCl]. Next, 1.5 mL of 100 µg/mL of purified *Y. pestis* F1 antigen (obtained from the CDC in Denver, Colorado) in 0.1 M NaHCO_3_ (pH 8.6) was incubated at 4 °C overnight in a 35 × 10 mm polystyrene dish (code 25060-60; Corning, New York, NY, USA). The *Y. pestis* F1-antigen-coated dish was blocked for 2 h at 4 °C with blocking buffer [0.1 M NaHCO_3_ (pH 8.6), 5 mg/mL bovine serum albumin (BSA), and 0.02% NaN_3_], and then washed six times with TBS containing 0.5% Tween 20 (TBS/T). After washing six times with TBS/T, 10 µL (2 × 10^11^ phage) of the pH.D.-12 library (#E8100S) was dissolved in 1 mL 0.5% TBS/T, and the mixture was added to the dish coated with F1. The F1-coated dish with the phage mixture was then incubated for 2 h at 4 °C. After incubation, it was washed six times with 0.5% TBS/T. The bound phage was eluted with 1 mL elution buffer (0.2 M glycine-HCl (pH 2.2) and 1 mg/mL BSA) for 10 min. The eluate was neutralized with 150 µL 1 M Tris-HCl (pH 9.1). The phage titers of serial dilutions of phage eluates were determined by plating on lawns of *E. coli* ER2738, as described below in the phage tittering section. 

### 4.4. Phage Amplification

Amplification of the eluted phage from each round of panning, as described above, was initiated by the addition of 0.2 mL of an exponentially growing culture of *E. coli* ER2738 cells (absorbance at 550 nm = 1.8) in 20 mL LB medium supplemented with tetracycline (20 µg/mL) for 8 h at 37 °C with shaking, at 225 revolutions per minute (rpm). Afterward, the culture was centrifuged at 10,000 rpm for 10 min. The supernatant was re-centrifuge. The resulting phage was purified from the supernatant by precipitation in 20% (*w/v*) polyethylene glycol-8000 and 2.5 M NaCl. The upper 80% (16 mL) of the resulting supernatant was added to another fresh tube. Next, 2.7 mL of PEG/NaCl (20% polyethylene glycol-8000 and 2.5 M NaCl) was added and allowed to stand on ice for 15 min. The precipitate was collected by centrifuging at 10,000 rpm at 4 °C (15 min). Finally, the pellet was dissolved in 1 mL of phosphate-buffered saline (PBS). The supernatant was transferred to a new 1.5 mL microfuge tube and spun for 5 min to pellet the residual cells. The supernatant was transferred to a fresh 1.5 mL microfuge tube and reprecipitate with 2.7 mL of PEG/NaCl, where it was incubated on ice for another 15 min. The phage was then precipitated by centrifugation at 10,000 rpm for 10 min at 4 °C and resuspended in 200 µL PBS. The final phage stock was stored at 4 °C in PBS. The final eluate was tittered to give well-separated colonies so that individual colonies could be propagated and analyzed. The procedure for panning, washing, elution, and amplification was repeated three times.

### 4.5. Phage Titering

To begin, 5 mL of LB (with 20 µg/mL tetracycline) medium was inoculated with ER2738 and incubated at 37 °C with shaking until growth reached the mid-exponential phase. Agarose Top preparations (LB with 7.5 g/L Bacto-Agar) were melted in a microwave and kept in sterile culture tubes (3 mL each) at 50 °C. Next, the LB/IPTG/XGal plates (LB with 15 g/L Bacto-Agar, 50 mg/L IPTG, and 40 mg/L XGal) were prewarmed at 40 °C. A 1:10 serial dilution of phage was prepared in LB medium, after which 10 μL of each dilution was added to 0.2 mL of ER2738 culture upon reaching the mid-exponential phase, vortexed, and incubated at room temperature for 5 min. This solution was then added to the Agarose Top preparations, vortexed, and poured over the LB/IPTG/XGal plates. The plates were then allowed to cool and were incubated overnight at 37 °C. The next day, the plaques were counted and multiplied by the dilution factor to reach the desired phage titer in plaque-forming units (pfu) per 10 μL.

### 4.6. Final Phage Preparation and DNA Extraction

After tittering the eluate of the 3rd round of biopanning, isolated phage clones were amplified. Individual phage clones (blue colonies) were grown from the clones that bound to the *Y. pestis* F1 antigen and single-stranded DNA through the following procedure. An overnight culture of the host (ER2738) strain was diluted at 1:200 in LB/Tet. The phage plaques were tooth-picked and transferred to 1 mL aliquots of a 1:200 dilution of a fresh overnight culture of *E. coli* ER2738 and placed in 13 × 100 mm glass tubes. The *E. coli* ER2738 were then grown with shaking for 8 h at 37 °C in a water bath, after which the culture was transferred to 1.5 mL microfuge tubes. This was then microcentrifuged for 5 min at 15,000 rpm. The upper 80% of the supernatant was transferred to a fresh 1.5 mL microfuge tube, where half (400 μL) of the supernatant was kept as the final phage preparation while the other half was used to extract DNA for sequencing. 

Next, 200 μL of 20% PEG with 2.0 M NaCl was added and allowed to sit for 10 min at room temperature. This solution was microcentrifuged for 10 min at 15,000 rpm and the supernatant removed. The pellet was resuspended in 100 μL iodide buffer (10 mM Tris-HCl, 1 mM EDTA, 4 M NaI, and pH 8.0) and precipitated with 250 μL of 95% ethanol. The mixture was incubated at room temperature for 10 min, after which it was remicrocentrifuged for 10 min at 15,000 rpm, with the supernatant discarded. The pellet was washed with 500 μL of 70% ethanol and dried briefly under vacuum. The pellet was then resuspended in 30 μL of sterile water. The phage DNA sequences were determined at the Center for Biotechnology and Genomics Core Facility (TTU, Lubbock, TX, USA) using the -96 gIII sequencing primer (New England Biolabs #1259). The N-terminal amino acid sequences of the gene III products of the selected display phage were deduced from their DNA sequences.

### 4.7. Phage Binding Analysis by the Spun Cell ELISA

In order to identify the phages that recognize the F1 antigen on the surface of a bacterial cell, we used the Spun-Cell ELISA assay. This assay was employed to target the portion of the F1 antigen that is displayed on the surface of an *E. coli* cell. The procedure was carried out as previously described by Benhar et al. [19]. Briefly, the *E. coli* XL1-blue parent strain and the *Y. pestis* F1-antigen-expressing *E. coli* XL1-blue/pYPR1 strain were grown overnight at 37^o^C in LB medium supplemented with an appropriate antibiotic. Bacterial cultures were diluted to an OD 600 nm~0.2 (1 × 10^8^ cells/mL). The cell culture suspension was then cleared by centrifugation. The cell pellet was then washed twice with PBS and resuspended in 0.2% TBS/T (Tris-buffered saline (TBS; pH 7.4) and 0.2% Tween 20). A 1 mL aliquot of the bacterial dilution was transferred to each 1.5 mL microfuge tube for analysis of the surface display of the antigen. Phage clones from the previous selection process (1 × 10^11^) were added to each 1.5 mL microfuge tube, and the mixture incubated at room temperature for 1 h. Cells were then centrifuged and washed. For detection of phage binding, we used an HRP-conjugated anti-M13 antibody (Amersham Pharmacia Biotech, # 27-9420-01) that was diluted to 1:5000 in blocking buffer. The cell mixtures were incubated for one hour on ice and washed five times with 0.2% TBS/T by repeated centrifugation and resuspension. Afterward, 100 µL of HRP 3,3′,5,5′-tetramethylbenzidine (TMB) substrate (Sigma, St. Louis, MO, # T0440) was added to each 1.5 mL microfuge tube and the reaction allowed to stand for 10 min at room temperature for detection. The color development was terminated with 100 µL Stop Reagent for TMB Substrate (Sigma, # S5814). The absorbance was recorded at 450 nm.

### 4.8. Methodology for Labeling Phage with Selenium

A phage clone, phage #8, chosen as the best binder from the Spun-Cell ELISA assay, was amplified in 200 mL LB-Tet (20 µg/mL) medium for 8 h by the addition of 2 mL of the *E. coli* ER2738 strain from an overnight culture. The resulting phage was then purified from the supernatant (Ph.D. Phage Display Libraries Instruction Manual—NEB #E8100S, E8101S, E8110S, E8111L, E8120S). The amplified eluate was then reamplified again until the volume of 1–5 mL (~1 × 10^13^ phage/mL) was reached. This solution would then be used in the covalent attachment of a selenium compound. The selenium compound (cyanatoseleno-acetic acid; NCSeCH_2_COOH; 16.4 mg) was dissolved in 1 mL of MES (2-(*N*-morpholino)ethanesulfonic acid) buffer in a 10-mm-diameter tube. Next, 22 mg of N-hydroxysulfosuccinimide (Sulfo-NHS) and 20 mg 1-ethyl-3-(3-dimethyl aminopropyl) carbodiimide (EDC, Pierce Chemical, Rockford, IL, USA) were added to the solution.

The reaction was then carried out with stirring at room temperature for 1 h. Next, 10 µL of the reaction mixture was added to every 1 mL phage (~1 × 10^13^ phage/mL) and stirred at room temperature for 30 min. The mixture was then transferred to a membrane tubing (MWCO (6-8000), Spectrum) and dialyzed in 5 L of water, which was changed every 2 hours at least 5 times. This process was done to eliminate unbound selenium compounds and other excess chemicals. Then, 1 mL of seleno-phage was purified from the supernatant by precipitation with 1/6 volume of PEG/NaCl (20% polyethylene glycol-8000 and 2.5 M NaCl). The precipitate was collected by centrifuging at 10,000 rpm at 4 °C (15 min). Finally, the pellet was dissolved in 1 mL of PBS. This seleno-phage stock was used in the killing assay.

To determine the presence of selenium on phage #8, 5 mL of dialyzed seleno-phage (~1 × 10^13^ phage/mL) was precipitated with 2.7 mL of PEG/NaCl (20% polyethylene glycol-8000 and 2.5 M NaCl). The pellet was then dissolved in 200 μL PBS. Next, 50 μL was used in the chemiluminescent assay to confirm the presence of selenium. To determine the presence of selenium on peptide #8, 1 mg of seleno-peptide #8 was dissolved in 1 mL of water. Finally, 100 μL of the seleno-peptide #8 solution was used in a quantitative fluorescent assay described elsewhere [16].

### 4.9. Bacterial Killing Assays with the Selenium-Labeled Phage and Peptides

The antibacterial activity of the phage and peptides covalently labeled with selenium was determined. The overnight *E. coli* XL1-blue and *E. coli* XL1-blue/pYPR1 bacterial cultures were diluted to the optical density of 0.2 (~10^8^ bacteria per mL) at 600 nm (A_600nm_), and cells were collected by centrifugation and washed twice with PBS. The cells were resuspended in the same volume of PBS, and aliquots equivalent to 1 mL were transferred to 1.5 mL microfuge tubes. The seleno-phage or seleno-peptides were added to a bacterial suspension, with a multiplicity of infection (MOI) of 1000:1 (phage per bacterium) or 10 µM, respectively. In designated tubes, glutathione was added to a final concentration of 150 or 300 µM, and the mixtures were incubated at room temperature. In the other experiments, extra oxygen was supplied from air by an air pump (Pipet Aid, Drummond Scientific Co. #262). Briefly, a flexible airline tube was connected to an air pump. The flexible airline tube was then connected to multiway gang valves. Each outlet from these multiway gang valves was plugged with a needle (B-D, #305185). The speed of flowing air was adjusted through the valves of the above multiway gang valves so that gentle bubbles were observed. At different time intervals, samples were collected, and serial dilutions (1:100) were performed. Viable cell counts were determined by plating serial dilutions on LB plates, with or without appropriate antibiotics, after 24 h of incubation at 37 °C.

### 4.10. Peptide Synthesis

The peptides, which represented the sequences obtained from the phage libraries, were synthesized on a small scale. Briefly, the synthesis of peptides was started from carboxy-terminal amino acid. First, 1 g resin (sometimes with the first amino acid on it) was transferred by DMF (dimethylformamide) to the peptide synthesizer and allowed to stand in the DMF for 30 min. The DMF was removed completely under vacuum. Second, 10 mL of 20% piperidine in DMF was added, and the mixture bubbled with N_2_ for 5 min. The solution was then drained, after which 10 mL of more piperidine solution was added, and the mixture bubbled for 20 min. The mixture was drained thoroughly and washed 3 times with DMF and 2 times with isopropanol. A small amount of the solution was used in a Ninhydrin test (1,2,3-indantrione monohydrate or triketohydrindene hydrate) to detect free amino groups in the presence of blue color. 

The rest of the beads were washed three more times with DMF. Two equivalents of amino acid (in 7 mL DMF) were added and bubbled with nitrogen gas. Two equivalents of PyBop (in 3 mL DMF) and four equivalents of pure DIEA (N N-diisopropylethylamine) were then added and bubbled with nitrogen gas for 1 h. The mixture was then drained and washed three times with DMF. A small amount of the beads was used in the Ninhydrin test, which produced a yellow color. The Fmoc (9-Fluorenylmethoxycarbonyl) group was removed from the amine terminal of a growing peptide chain in basic conditions (usually 20% piperidine in DMF). The procedure was repeated until the last desired amino acid was added. The mixture was then washed three times with DMF, three times with CH_2_Cl_2_, and three times with methanol. The mixture was then drained thoroughly. The resin was transferred to a vial, freeze-dried overnight, and then stored in the freezer. The peptides were then prepared and dissolved at 1 mg/mL concentration in water (pH = 8.0).

### 4.11. Peptide #8 Structure and Amino Acid Sequence Analysis

The synthetic peptide #8 was purified by high performance liquid chromatography (HPLC). The purification was achieved by reverse-phase column with linear gradients of 0.1% TFA in water and 0.075% TFA in CH_3_CN at a 1.5 mL /min flow rate; the gradient range (20% CH_3_CN to 100% CH_3_CN) vs. gradient time (40 min) vs. gradient steepness (2%/min). The molecular weight was confirmed by mass spectroscopy (MW = 1307.692, Colorado State University, Fort Collins CO). The amino acid sequence of peptide #8 was determined and confirmed by a protein sequencing system at the Center for Biotechnology and Genomics Core Facility (TTU, Lubbock, TX, USA). The peptide’s amino acid sequence was also confirmed by two-dimensional NMR spectroscopy (2D-NMR).

### 4.12. Seleno-Peptide Synthesis

For this synthesis, a BrCH_2_CH_2_CO-group is coupled to the terminal amino residue of the peptide on the resin obtained above. Twelve equivalents of BrCH_2_CH_2_COOH in dry CH_2_Cl_2_ were added and allowed to dissolve. The reaction flask was closed, and the solution was cooled with ice water. A solution of DCC (six equivalents) in dry CH_2_Cl_2_ was added and stirred for 20 min. The white solid was filtered off, and then the solvent was removed by rotary evaporation to obtain the anhydride. First, resin and DMF were mixed and allowed to stand for 30 min, after which the resin was drained thoroughly. The anhydride was dissolved in DMF, and the solution was added to the resin. The mixture was bubbled with nitrogen gas for an hour. The mixture was then washed three times with DMF, three times with CH_2_Cl_2,_ and three times with methanol. The solvents were drained thoroughly. The resin was then transferred to a vial, processed by freeze-drying overnight, and stored in the freezer. The peptide was then cleaved from the resin. The dry resin was placed in a vial, and TFA (trifluoroacetic acid) added at the ratio of TFA/TIS (triisopropyl-silane)/water of (95:2.5:2.5), using 15 mL/g resin. The mixture was allowed to stand at room temperature with occasional swirling for 2 h. The resin was then removed by filtration under reduced pressure and washed twice with TFA.

The TFA was evaporated by a rotary evaporator, and an 8- to 10-fold volume of cold ether was added (dropwise) to the filtrates. The suspension was then transferred to a clean 1.5 mL microfuge tube, sealed, and centrifuged. Ether was decanted from the tube. An additional 8- to 10-fold volume of cold ether was added to wash scavengers, followed by centrifugation. The residual solid was dissolved in a CH_3_CN/H_2_O (50/50) mixture and then lyophilized. Next, 20 mg of crude bromo-peptide and 20 mg potassium selenocyanate were dissolved in 1 mL of DMF. The reaction was allowed to proceed at room temperature for 24 h. Then, 1 mL of water and 20 mg of ammonium chloride were added to quench the excess seleno-cyanate. The seleno-cyanate peptide was then purified by reverse-phase HPLC. The lyophilized peptide was stored by freezing at −20 °C. All seleno-peptides synthesized were tested for superoxide radical generation capability by a chemiluminescent assay (see below). In addition, seleno-peptides were prepared at 1 mg/mL concentration in water (pH = 8.0). The conformation was studied and compared to that of peptides without selenium attachment by circular dichroism.

### 4.13. Chemiluminescent (CL) Assay

A chemiluminescent assay was used to determine the activity of selenium on the phage and peptides. The control chemiluminescent (CL) assay cocktail, without substrates or GSH (reduced glutathione), was made using 0.05 M sodium phosphate buffer (pH = 7.4) and 20 μL lucigenin/mL from a stock solution of 1.0 mg/mL lucigenin in distilled water. The assay cocktail with thiol contained GSH (1.0 mg/mL); 50 µL of seleno-phage or phage (10^7^ phage/mL) or 50 μL of seleno-peptide or peptide (10 µg/mL) were added to 500 μL test aliquots of the control or thiol-containing assay cocktail. Chemiluminescent (CL) data were recorded in integrated units over a period of 5 min.

### 4.14. Competitive Inhibition ELISA

To check the binding of the peptide to target proteins, we employed a competitive inhibition ELISA assay. The *E. coli* XL1-blue/pYPR1 and XL1-blue strains were grown overnight at 37 °C under the conditions described above. Bacterial cultures were diluted to OD_600nm_ ~0.2 (~10^8^ cfu/mL), and 1 mL of the bacterial dilution was transferred to each microcentrifuge tube. The pYPR1 and XL1-blue cells were used to determine the binding competition between peptide #8 and the F1 antibody for the F1 antigen on pYPR1 cells. The pYPR1 cells were then first incubated with either peptide #8 (1 µM) or the F1 antibody (0.25 µg/mL) for an hour at 4 °C. The cells were centrifuged and washed. The F1 antibody (0.25 µg/mL) and peptide #8 (1 µM) were then added, whereby the suspensions were incubated at 4 °C for an hour. Cells were centrifuged and washed 5 times. The F1 antibody was detected using an anti-mouse monoclonal IgG antibody that was HRP-conjugated (1:3000). The cell mixtures were incubated for one hour on ice and washed five times with 0.2% TBS/T by repeated centrifugation and resuspension. Next, 100 μL of 3,3′,5,5′-tetramethylbenzidine (TMB) substrate (Sigma, # T0440) was added to each 1.5 mL microfuge tube. The reaction was allowed to stand for 10 min at room temperature for detection. The color development was terminated with 100 μL Stop Reagent for TMB Substrate (Sigma, # S5814), and the absorbance was recorded at 450 nm.

### 4.15. Extraction of F1 Antigen from E. coli

The extraction of the F1 antigen was performed as previously described by Simpson et al. [20]. The *E. coli* cells carrying pYPR1 were grown overnight in 20 mL LB medium supplemented with carbenicillin (100µg/mL) and then harvested by centrifugation using 10,000 rpm for 5 min in a Beckman rotor. Next, 80% of the supernatant was transferred to a fresh tube, while the remaining supernatant was transferred to another tube. Above the cell pellet was a less dense, flocculent material, which was recovered with the supernatant. This material was solubilized in SDS-PAGE buffer, boiled for 5 min, and loaded onto a 14% preparative SDS-polyacrylamide gel. The cell pellet was washed twice gently in 2 mL PBS, repelleted by centrifugation, and resuspended in 2 mL PBS. Following electrophoresis, the gel was stained with water-based Coomassie Blue R270 to identify the 14 to 17 kDa band, which corresponded to the F1 antigen. The presence of the F1 antigen in the supernatant was subsequently confirmed by immunoblot. 

### 4.16. SDS-Page and Western Blot Procedure

The molecular weight and relative amount of F1 antigen produced by recombinant *E. coli* pYPR1 were determined by SDS-polyacrylamide gel electrophoresis (SDS-PAGE) and Western blot. The pYPR1 was grown from a single colony in 20 mL LB medium supplemented with 100 µg/mL carbenicillin for 24 h and then harvested by centrifugation, using 10,000 rpm for 5 min in a Beckman rotor. Approximately 80% of the supernatant was transferred to a 1.5 mL fresh tube. The remaining supernatant was transferred to another tube. Then, 1 mL of the supernatant factions was treated with 100 μL trichloroacetic acid (TCA) and incubated on ice for an hour. The TCA-treated factions were then centrifuged for 5 min and the supernatants discarded. The pellets were washed with TBS [50 mM Tris-HCl (pH 7.5) and 150 mM NaCl]. The pellets were resuspended in 100 μL sterile water and 100 µL 4X sample buffer. 

For supernatant samples without TCA treatment, 100 μL supernatant was transferred to a 1.5 mL fresh tube, to which 100 μL 4X sample buffer was added. The purified F1 antigen sample was prepared by mixing 50 μL from purified F1 stock solution (1 mg/mL) with 50 μL sterile water and 100 µL 4X sample buffer. Cell pellet samples were prepared by suspending the pellets in 2 mL PBS. Next, 100 μL was then transferred to a 1.5 mL microfuge tube, to which 100 µL 4X sample buffer was added. The XL1-blue sample was prepared using 1 mL of overnight culture, which was transferred to a 1.5 mL microfuge tube. The cells were centrifuged, and the supernatant was discarded. The cell pellet was resuspended in 100 μL sterile water and 100 µL 4X sample buffer. All the samples were boiled for 5 min, and 20 μL of the denatured sample was loaded and run on a 14% SDS-PAGE. The resulting gel proteins were transferred to a PVDF membrane (BIO-RAD, #14098) using the Trans-Blot SD Semi-Dry Transfer Cell (Bio-Rad, #221BR 17035). The membrane was blotted with blocking solution (2% BSA and 7.5% nonfat dry milk, 0.2% Tween-20 in TBS (60 mg/mL NaCl, 1.21 mg/mL Tris-base, and pH = 7.4)) for an hour with gentle rocking at room temperature. 

The membrane was then washed 5× in wash buffer (0.2% Tween-20 in TBS), 10 min with gentle rocking per wash. Afterward, the primary antibody (diluted 1:4000 mouse monoclonal IgG F1 antibody (1 mg/mL) in wash buffer with 0.25% BSA and 2% nonfat dry milk) was added and rocked gently for 1 h. The membrane was then washed 5x in wash buffer (0.2% Tween-20 in TBS), 10 min with gentle rocking per wash. Then, the secondary antibody (diluted 1:3000 anti-mouse monoclonal IgG antibody (1 mg/mL) in wash buffer with 0.25% BSA and 2% nonfat dry milk) was added and rocked gently for 1 h. The membrane was then washed 5 more times in wash buffer, then covered in Pierce Super Signal West Pico Chemiluminescent Substrate (5 mL peroxide solution and 5 mL luminol/enhancer solution) (#34080) for one minute. The membrane was then exposed to Blue Sensitive Autoradiographic Film (Marsh Bio Products #75590) for 3 min and developed.

### 4.17. Statistical Analysis

Statistics were calculated using InStat (Graph Pad Software, San Diego, CA). The *p*-values were calculated according to the paired *t*-test, where a significant value was *p* < 0.5.

## 5. Conclusions

These results show that covalently selenium-labeled peptides and viruses (i.e., bacteriophages) can be selectively made to bind to the surface of pathogenic bacteria and then kill them through the generation of superoxide on their surface. The attachment of a selenium-labeled phage or peptides to the surface of a bacteria was sufficient for bacterial killing; however, if the phage or peptides did not attach to the cell, no killing was observed. These seleno-peptides or selenium-labeled display phage should provide a highly specific and safe means for rapid protection from known bacterial pathogens without affecting other bacteria. This approach to drug development, using the site-specific, localized generation of superoxides, is remarkably similar to the mechanism whereby lymphocytes, following phagocytosis of bacteria, generate superoxides from membrane-bound NADPH oxidase to lyse bacteria in the phagolysosome.

## Figures and Tables

**Table 1:** *Y. pestis* F1-antigen-specific phage-display peptide sequences.

Phage #	Peptide Sequence											
1	Ser	Phe	Ser	Leu	Lys	Pro	His	Ala	Ser	Leu	Ile	Arg
2	Gly	Pro	Asn	Lys	Phe	Ser	Leu	Met	His	Leu	Phe	Ser
3	Ser	Phe	Ser	Leu	Ser	Ser	Tyr	Ser	Ala	Leu	Leu	Trp
4	Lys	Phe	Ser	Leu	Ser	Pro	His	Thr	Ala	Trp	Phe	Leu
5	Lys	Leu	Ser	Leu	Asn	Pro	His	Phe	Met	Phe	Gln	Ser
6	Phe	Ser	Leu	Lys	Asn	Pro	Thr	Ile	Ala	Asn	Thr	Met
7	Leu	Ile	Ser	Val	Glu	Pro	Ala	Ser	Leu	Ser	Ala	His
8	Ser	Ser	Leu	Thr	Leu	Ala	Pro	Phe	Ser	Trp	Ser	Leu
9	Gly	Pro	Trp	Phe	Ser	Leu	Arg	His	Leu	Ser	Pro	Gln
10	Ser	His	Ser	Trp	Phe	Arg	Val	Asn	Thr	Leu	His	Leu
11	Gly	Trp	Phe	Ser	Thr	Pro	Leu	Lys	Trp	Arg	Met	Gln
12	Ser	Asn	Phe	Thr	Leu	Pro	Phe	Leu	Lys	Thr	Phe	Arg
13	Ser	Trp	Phe	Thr	Leu	His	Asn	Leu	Pro	Asn	Arg	Pro
14	Asn	Phe	Ser	Ile	Asn	Pro	Arg	Met	Met	Trp	Pro	Val
15	Ser	Trp	Phe	Pro	Phe	Lys	Gln	Ser	His	Arg	Pro	Arg

## Data Availability

Data supporting the reported results can be found archived in the TTUHSC Medical School Library.

## Supplementary Materials

The following are available online at https://www.mdpi.com/article/10.3390/antibiotics10060611/s1, Figure S1: Sequence Motif Consensus Sequence Analysis from Peptide Sequences using MEME (https://memesuite.org/meme/tools/meme, accessed on 20 May 2021); Figure S2: Discovered Motifs from Peptide Sequences using MEME (https://memesuite.org/meme/tools/meme, accessed on 20 May 2021); Figure S3: Frequency of Amino Acids from Peptide Sequences using MEME (https://memesuite.org/meme/tools/meme, accessed on 20 May 2021); Figure S4: Settings Used for Analysis of Peptides Using MEME (https://memesuite.org/meme/tools/meme, accessed on 20 May 2021).
