# Supplementary material for: The Use of an Organo-Selenium Peptide to Develop New Antimicrobials That Target a Specific Bacteria
Source: Antibiotics (Basel). 2021 May 21;10(6):611. doi: 10.3390/antibiotics10060611 (PMC8224008; doi:10.3390/antibiotics10060611)
Supplement: Supplementary file 1 [file antibiotics-10-00611-s001.zip › antibiotics-1165193-supplementary.pdf]

The following supplements refer to Table 1 in the manuscript.

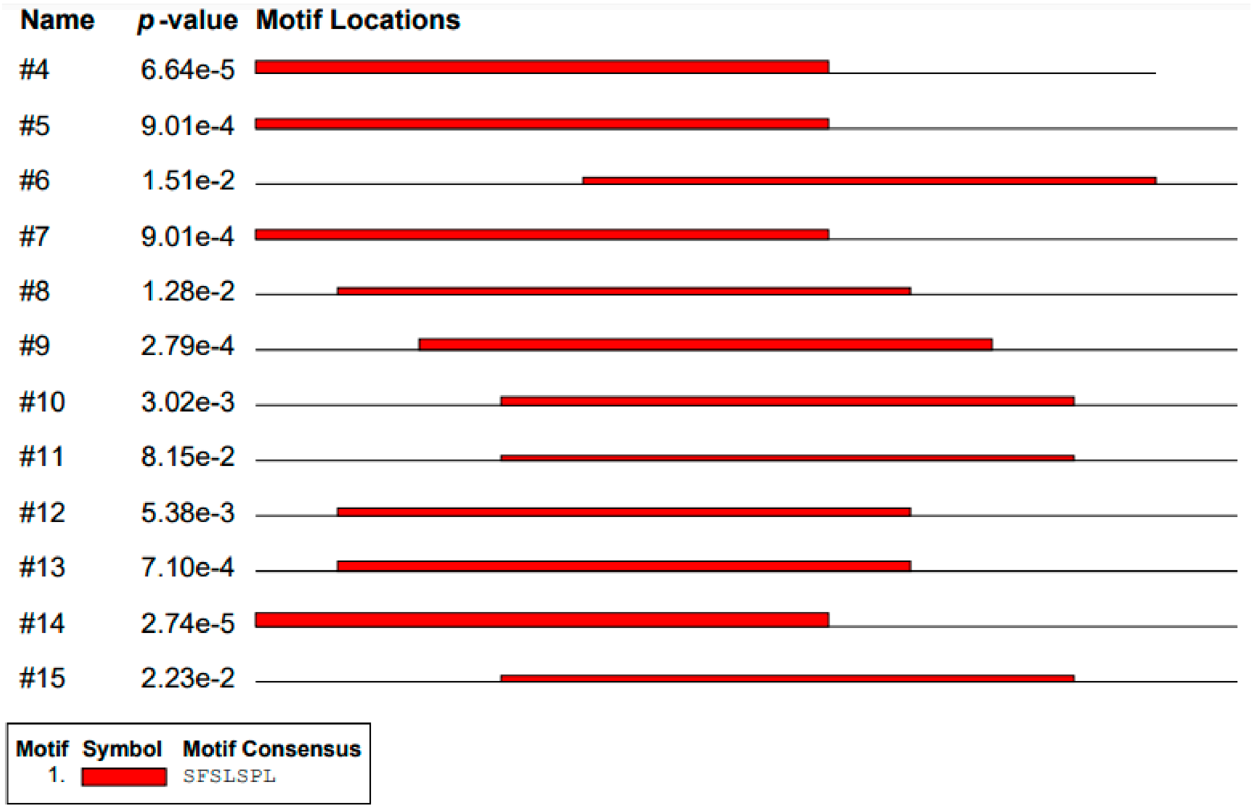

Figure S1. Sequence Motif Consensus Sequence Analysis from Peptide Sequences using MEME (https://meme-suite.org/meme/tools/meme). The thickness of the bar corresponds to probability of sequence overlap. And the length corresponds to the location.

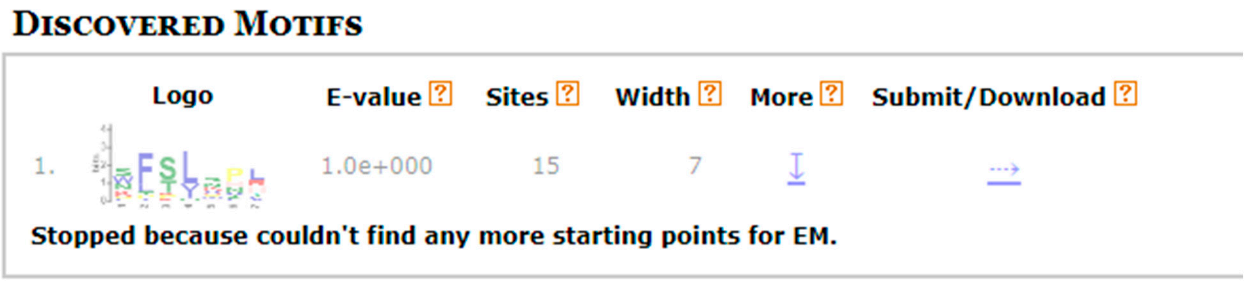

Figure S2. Discovered Motifs from Peptide Sequences using MEME (https://meme- suite.org/meme/tools/meme). See Motif consensus sequence above in Supplemental Figure 1.

Role	Source	Alphabet	Sequence Count	Total Size
Primary Sequences	sequences.fa	Protein	15	177

Background Model

Source: built from the (primary) sequences

Order: 0

	Name	Freq.	Bg.
A	Alanine	0.0395	0.0395
C	Cysteine	0	0.0000282
D	Aspartic acid	0	0.0000282
E	Glutamic acid	0.00565	0.00567
F	Phenylalanine	0.113	0.113
G	Glycine	0.0169	0.017
H	Histidine	0.0565	0.0565
I	Isoleucine	0.0226	0.0226
K	Lysine	0.0452	0.0452
L	Leucine	0.141	0.141
M	Methionine	0.0339	0.0339
N	Asparagine	0.0565	0.0565
P	Proline	0.096	0.096
Q	Glutamine	0.0226	0.0226
R	Arginine	0.0508	0.0508
S	Serine	0.175	0.175
T	Threonine	0.0508	0.0508
V	Valine	0.0169	0.017
W	Tryptophan	0.0508	0.0508
Y	Tyrosine	0.00565	0.00567

Figure S3. Frequency of Amino Acids from Peptide Sequences using MEME (https://meme- suite.org/meme/tools/meme).

Other Settings

**Motif Site Distribution**	ZOOPS: Zero or one site per sequence
**Objective Function**	E-value of product of p-values
**Starting Point Function**	E-value of product of p-values
**Site Strand Handling**	This alphabet only has one strand
**Maximum Number of Motifs**	3
**Motif E-value Threshold**	no limit
**Minimum Motif Width**	6
**Maximum Motif Width**	12
**Minimum Sites per Motif**	2
**Maximum Sites per Motif**	15

Show Advanced Settings

Figure S4. Settings Used for Analysis of Peptides Using MEME (https://meme- suite.org/meme/tools/meme).
